# Evaluation of fecal microRNA stability in healthy cats

**DOI:** 10.1111/vcp.12757

**Published:** 2019-06-26

**Authors:** Janne G. Lyngby, Annemarie T. Kristensen, Merete Fredholm, Lise N. Nielsen, Susanna Cirera

**Affiliations:** ^1^ Department of Veterinary Clinical Sciences, Faculty of Health and Medical Sciences University of Copenhagen Copenhagen Denmark; ^2^ Department of Veterinary and Animal Sciences, Faculty of Health and Medical Sciences University of Copenhagen Copenhagen Denmark

**Keywords:** biomarker, cat, feces, miRNA, qPCR, stability

## Abstract

**Background:**

Gastrointestinal (GI) cancer accounts for 14% of feline malignancies. There is a great need for reliable noninvasive diagnostic biomarkers to reach a timely diagnosis and initiate treatment. Fecal microRNAs (miRNAs) could be such a biomarker and have shown great potential in colorectal screening in people but have yet to be investigated in cats.

**Objectives:**

We aimed to evaluate the presence and stability of feline fecal miRNA under different storage conditions (room temperature [RT], 4, and −20°C) and to evaluate the expression levels of specific fecal miRNAs collected on three separate days (days 1, 4, and 7) in healthy cats.

**Methods:**

Healthy cats were prospectively recruited. Fecal samples were collected, aliquoted, and stored for 24 hours at RT and then transferred to −20°C, stored for 24 hours at 4°C and then transferred to −20°C, or were immediately placed at −20°C on day 1 or at −20°C on days 4 and 7 postcollection. Expression of 22 miRNAs was investigated using quantitative real‐time PCR.

**Results:**

Ten miRNA assays worked well, and one, let‐7b, was used for normalization. No differences in miRNA expression were seen between the three storage temperatures for the nine miRNAs investigated. Only miR‐26a showed a significant increase in expression between samples of days 1 and 7. The rest of the miRNAs levels were stable over time.

**Conclusions:**

Fecal miRNA can be isolated from healthy cats. The expression was stable at different temperatures and for most of the miRNAs over time. Prospective studies evaluating fecal miRNA as biomarkers in cats with GI neoplasia are warranted.

## 1 INTRODUCTION

Gastrointestinal (GI) cancer in cats accounts for around 14% of feline malignancies,^1^ and with advances in veterinary diagnostics and willingness to diagnose and treat feline patients, the importance of reaching a diagnosis to initiate a correct treatment is as important as ever.^2^ Lymphoma is the most common GI cancer in cats followed by adenocarcinoma and mast cell tumors.^2^ Of the lymphomas, approximately 75% are small cell lymphoma.^3^ The preferred treatment and prognosis vary significantly with the different cancer types, hence reaching a diagnosis will determine the best therapeutic option for a patient.

Unfortunately, the clinical signs of GI cancer are nonspecific and not significantly different from those of other chronic enteropathies. This includes weight loss, vomiting, diarrhea and/or hyporexia/anorexia, which makes it difficult to differentiate one disease from another.^4^, ^5^ To rule out extraintestinal disease and reach a diagnosis, a systematic work‐up is required. This can include a hemogram, serum biochemical panel, basal cortisol/adrenocorticotropic hormone stimulation testing, pancreatic lipase immunoreactivity (PLI), trypsin‐like immunoreactivity (TLI), cobalamin, and folate. Moreover, urine and fecal testing, abdominal ultrasound, and GI biopsies with histopathologic evaluations are used to differentiate between various chronic enteropathies and provide a correct diagnosis.^4^ This can be costly and potentially invasive, resulting in some clients and veterinarians electing to initiate therapeutic trials or euthanize a patient without a firm diagnosis. Even if a diagnosis is reached, the time from initial primary complaint to diagnosis is often prolonged, thereby delaying correct treatments and potentially resulting in disease progression and decreased survival times. Thus, fast, reliable, and noninvasive diagnostic tests to differentiate GI cancer from other chronic enteropathies in cats is needed.

MicroRNAs (miRNAs) are small noncoding RNAs that have important roles in gene regulation. They exert their function at the post‐transcriptional level by binding to the complementary mRNA strands and thereby inhibiting translation and/or triggering  mRNA degradation. MicroRNA regulation is essential for many processes involved in cell division and maturation in many species and both health and disease.^6^ Recently fecal miRNA has been investigated as a noninvasive screening test for colorectal cancer (CRC) in humans.^7^ Our group has validated an assay for extraction of fecal miRNA from dogs and assessed its stability; however, no similar information exists in cats.^8^

Our objective was to evaluate the presence and stability of fecal miRNA under different storage conditions (room temperature [RT], 4, and −20°C) and to evaluate the expression levels of specific fecal miRNAs on three separate days (days 1, 4, and 7) in healthy cats. We hypothesized that reliable, stable, and specific fecal miRNA expression could be established in healthy cats.

**Table 1:** The microRNAs examined in this study

Assay	Forward primer	Reverse primer
miR‐15a	5′‐GCAGTAGCAGCACATAATGG‐3′	5′‐CCAGTTTTTTTTTTTTTTTACAAACCA‐3′
miR‐16	5′‐CAGTAGCAGCACGTAAATATTG‐3′	5′‐CAGTTTTTTTTTTTTTTTCGCCAA‐3′
miR‐21	5′‐GCAGTAGCTTATCAGACTGATG‐3′	5′‐GGTCCAGTTTTTTTTTTTTTTTCAAC‐3′
miR‐26a	5′‐GCAGTTCAAGTAATCCAGGATAG‐3′	5′‐GTCCAGTTTTTTTTTTTTTTTAGCCT‐3′
miR‐26b	5′‐CGCAGTTCAAGTAATTCAGGA‐3′	5′‐GGTCCAGTTTTTTTTTTTTTTTACCT‐3′
miR‐192	5′‐CAGCTGACCTATGAATTGACA‐3′	5′‐CCAGTTTTTTTTTTTTTTTGGCTGT‐3′
miR‐200a‐3p	5′‐CAGTAACACTGTCTGGTAACG‐3′	5′‐GGTCCAGTTTTTTTTTTTTTTTACATC‐3′
miR‐200c‐3p	5′‐AGTAATACTGCCGGGTAATG‐3′	5′‐GTCCAGTTTTTTTTTTTTTTTCCATC‐3′
let7a‐5p	5′‐GCAGTGAGGTAGTAGGTTGT‐3′	5′‐GGTCCAGTTTTTTTTTTTTTTAACTATAC‐3′
let7b‐5p	5′‐GTGAGGTAGTAGGTTGTGTG‐3′	5′‐GGTCCAGTTTTTTTTTTTTTTTAACCA‐3′
miR‐141‐3p	5′‐GCAGTAACACTGTCTGG TAAAG 3′	5′‐GGTCCAGTTTTTTTTTTTTTTTCCAT‐3′
miR‐1224‐5p	5′‐GGTGAGGACTCGGGAG‐3′	5′‐GGTCCAGTTTTTTTTTTTTTTTCCA‐3′
miR‐155	5′‐CGCAGTTAATGCTAATTGTG‐3′	5′‐CAGTTTTTTTTTTTTTTTCCCCTATC‐3′
miR‐194	5′‐AGTGTAACAGCGACTCCA‐3′	5′‐GTCCAGTTTTTTTTTTTTTTTCCAC‐3′
let‐7g	5′‐CGCAGTGAGGTAGTAGTTG‐3′	5′‐CAGGTCCAGTTTTTTTTTTTTTTTAAC‐3′
miR‐29b‐3p	5′‐CATCTTTGTATCTAGCACCATTTGAAAT‐3′	5′‐GGTCCAGTTTTTTTTTTTTTTTAACACT‐3′
miR‐378‐3p	5′‐AGACTGGACTTGGAGTCAG‐3′	5′‐CAGTTTTTTTTTTTTTTTGCCTTCTG‐3′
miR‐574	5′‐TGAGTGTGTGTGTGTGAGT‐3′	5′‐CAGGTCCAGTTTTTTTTTTTTTTTACA‐3′
miR‐194	5′‐AGTGTAACAGCGACTCCA‐3′	5′‐GTCCAGTTTTTTTTTTTTTTTCCAC‐3′
miR‐200b‐3p	5′‐ACAGTAATACTGCCTGGTAATG‐3′	5′‐GGTCCAGTTTTTTTTTTTTTTTCATC‐3′
miR‐23a‐3p	5′‐AUCACAUUGCCAGGGAUUUCCA‐3′	5′‐CGTCCAGTTTTTTTTTTTTTTTGGAA‐3′
miR‐20a	5′‐CAGTAGCAGCACGTAAATATTG‐3′	5′‐GTCCAGTTTTTTTTTTTTTTTCTACCT‐3′
miR‐92a	5′‐AGGTGTGTATAAAGTATTGCACTTGTCC‐3′	5′‐CAGGTCCAGTTTTTTTTTTTTTTTACAG‐3′

Forward and reverse primers were used.

Abbreviation: miR, microRNA.

## 2 MATERIALS AND METHODS

### 2.1 Patient recruitment and screening

This study was approved by the ethics committee of the Department of Veterinary Clinical Sciences, University of Copenhagen (#2017‐9) with informed client consent. The study was conducted during the summer of 2017.

Sixteen healthy adult privately owned cats were prospectively recruited for this research project. The cats were fed a commercially available wet and/or dry cat food, and no intervention was made to the diets on behalf of the study.

Each cat was fasted prior to examination, and a full physical examination was performed by one of the authors (JGL). Blood was collected for routine hematology, serum biochemistry panel, total thyroxin (tT4), and urine and feces were collected for a urinalysis and fecal flotation, respectively. The urine sample was collected from clean litterboxes with nonabsorbent litter following ethics approval. Cats were excluded if they weighed less than 2.5 kg, were fed a raw food diet, were found to have GI abnormalities on physical examination, had received any medications including anti‐inflammatory or immunosuppressive drugs within 6 weeks of the study start, or had been dewormed within a month of the study start.

### 2.2 Fecal collection, transportation, and storage

As part of the study, clients were provided an at‐home fecal collection protocol that included a written manual that was explained to the client and all materials needed for fecal collection.

**Figure 1:**
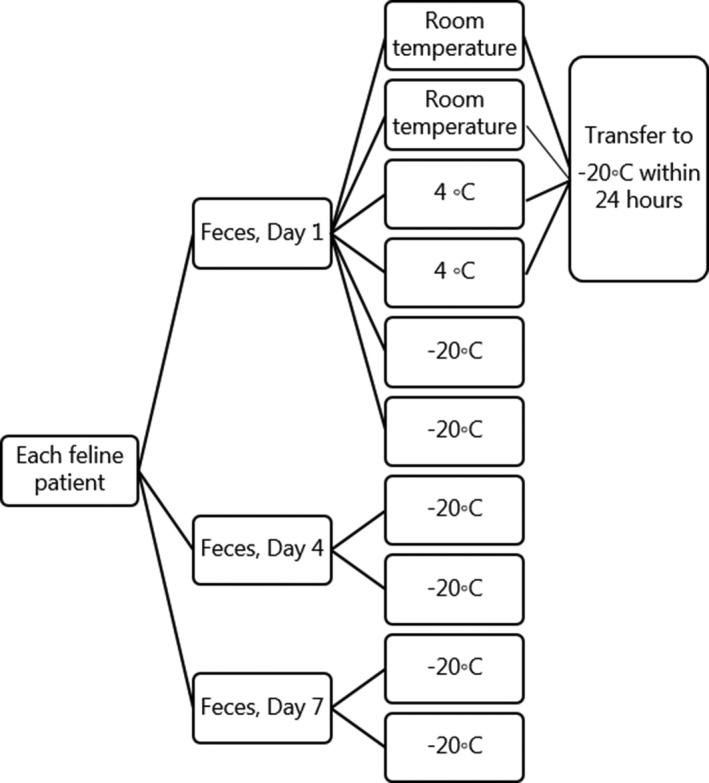
The protocol for fecal collection and storage from each feline patient. On day 1, the fecal sample was aliquoted and stored in duplicate at room temperature (RT), 4, and –20°C. The samples stored at RT and 4°C were transferred to –20°C after 24 h. On days 4 and 7, the samples were stored directly at –20°C

To assess miRNA stability and compare the effect of different storage temperatures, feces were collected on three different days—days 1, 4, and 7. Samples were collected from the litterbox as soon after defecation as possible and within one hour. Approximately one gram of each fecal samples was aliquoted into 2.0‐mL cryotubes in duplicate for each storage condition. See Figure 1 for a schematic presentation of the fecal collection and storage protocol. On day 1, two tubes were stored at RT (approximately 22°C) for 24 hours and then moved to –20°C, two cryotubes were stored at 4°C for 24 hours and then moved to −20°C, and two cryotubes were stored directly at −20°C (see Figure 1). To evaluate intra‐cat miRNA variation among the different collection days, two cryotubes were stored directly at −20°C on three different days (days 1, 4, and 7). The samples were stored at −20°C in different household freezers to represent at‐home collection. Within 1 month of fecal collection, all samples were collected in a cooling bag and transported on dry ice to the laboratory. All samples were evaluated upon arrival to ensure that they had not thawed and were stored at −80°C until further analyses, which was within 8 months.

### 2.3 RNA extraction

One hundred milligrams of feces was homogenized in 1000 µL RNase‐free water in M Tubes (Milteny, Bergisch Gladbach, Germany) on a *gentleMACS Octo* Dissociator (Milteny, Bergisch Gladbach, Germany). RNA was extracted with a miRNeasy kit (Qiagen, Hilden, Germany) by the addition of 200 µL homogenate to 1200 µL QIAzol buffer following the manufacturer's protocol without DNase treatment. The RNA concentration and the purity of each sample were measured on a NanoDrop1000 Spectrophotometer (Thermo Scientific, Hvidovre, Denmark).

### 2.4 cDNA synthesis

Three cDNA replicates for each sample were made using 100 ng of total RNA for each synthesis according to Balcells et al.^9^ Briefly, 1 µL 10× Poly(A) Polymerase buffer (New England Biolabs, Massachusetts, USA), 0.1 mmol/L ATP, 1 µmol/L RT‐primer (5′‐CAGGTCCACTTTTTTTTTTTTTTTVN; V = A, C, or G; N = A, C, G or T), 0.1 µmol/L deoxyadenosine triphosphate, 0.1 µmol/L deoxycytidine triphosphate, 0.1 µmol/L deoxyguanosine triphosphate, 0.1 µmol/L deoxythymidine triphosphate, 100 units MuLV Reverse Transcriptase (New England Biolabs), and 1 unit Poly(A) Polymerase (New England Biolabs) were mixed with the RNA in a final reaction volume of 10 µL. Negative controls were made by exclusion of Poly(A) Polymerase. cDNA samples were diluted eightfold before proceeding with qPCR.^9^

### 2.5 Selection of miRNA candidates

Seventeen miRNAs (miR‐1224, miR‐155, miR‐194, miR‐26b, miR‐200a‐3p, let‐7g, miR‐192, miR‐141‐3p, miR‐574, let‐7a, miR‐29b‐3p, let‐7b, miR‐378, miR‐200b‐3p, miR‐23a‐3p, miR‐15a, and miR‐200c‐3p), previously found to be among the 50 most expressed miRNAs in both human and mouse feces, were tested.^10^ Furthermore, miR‐16, miR‐20a, miR‐21, miR‐26a, and miR‐92a were included based on human fecal miRNA studies that were known to be expressed in feces.^7^ Specific miRNA primers were designed for each of the 22 miRNAs using the miRSpecific software^11^ as previously described (see Table 1).^9^, ^12^

### 2.6 Quantitative real‐time PCR

Quantitative real‐time PCR (qPCR) was performed in an M×3005 Pro qPCR system (Stratagene, California, USA) using the associated software. Quantifast SYBRGreen Master Mix (Qiagen) was used with PCR cycling conditions of 5 minutes at 95°C, 40 cycles of 10 seconds at 95°C, and 30 seconds at 60°C, followed by dissociation curve analysis of 1 minute at 95°C, 30 seconds at 55°C, and 1 minute at 95°C. To calculate the PCR efficiency for each assay, standard curves from a five‐time dilution series of a pool of all cDNA samples were performed.

The obtained cycle of quantitation (Cq) values were preprocessed using Genex Pro software (Multid, Gothenburg, Sweden). Briefly, Cq values were calibrated between plates for each assay, and PCR efficiency corrected. Replicates differing above two cycles from the two other replicates were excluded from the analysis. The data were normalized using the most stable miRNA (let‐7b) according to NormFinder and GeNorm^13^, ^14^ software programs. Subsequently, replicates were averaged for further analysis and relative quantities were calculated with respect to the least expressed sample in each assay. The data were log2 transformed before proceeding to statistical analysis. The qPCR experiments, as well as the data analysis, were all compliant with the Minimum Information for publication of quantitative real‐time PCR experiments (MIQE) guidelines.^15^

### 2.7 Statistical analyses

Log2‐transformed relative quantities for each miRNA were analyzed using linear mixed models, and using day temperature as an unbalanced interaction term and cats as random effects. Pairwise comparisons between combinations of day and temperature were carried out. A *P*‐value of <0.05 was considered significant. All statistical analyses were carried out using r (R Core Team, 2017) with the extension packages lme4 and multcomp. Figures and descriptive statistics were analyzed using GraphPad Prism 7 (GraphPad Software, San Diego, CA). Normality was assessed using the D'Agostino & Pearson normality test. Normally distributed values were reported as the mean ± standard deviation (SD).

## 3 RESULTS

### 3.1 Patient demographics

Sixteen healthy client‐owned cats were screened for inclusion. Seven cats were excluded for the following reasons: mild leukopenia, mild neutropenia, or other significant abnormalities on hemogram, biochemistry panel or urinalysis (n = 2); intestinal parasites (n = 1), diagnosed with feline idiopathic cystitis (n = 1), chronic kidney disease (n = 1), aortic murmur (n = 1), and incomplete fecal collection by the client (n = 1). A total of nine healthy cats were included in the study (see Table 2). The mean age was 60 months (SD: 38.5 months). There were six neutered males and three spayed females. The breeds included in the study were domestic shorthair (n = 3), Birma (n = 2), and an n = 1 of each of the following: ragdoll, British shorthair, and Bengal cats. All cats were fed commercially available wet and/or dry cat food and diets were not changed during the study period.

**Table 2:** Patient demographics of all healthy cats in this study

Cat ID	Age (mo)	Sex	Breed
Cat 1	35	MN	British shorthair
Cat 2	49	FS	DSH
Cat 3	121	MN	Birman
Cat 4	121	FS	Birman
Cat 5	59	MN	Ragdoll
Cat 6	14	MN	DSH
Cat 7	47	MN	Bengal
Cat 8	70	FS	Bengal
Cat 9	24	MN	DSH

Abbreviations: DSH, domestic shorthair; FS, female spayed; MN, male neutered.

### 3.2 miRNA detection

We successfully isolated fecal miRNA from healthy cats using the miRNeasy kit (Qiagen). The miRNeasy kit produced an average of 338 ± 179 ng/µL of total RNA per 100 mg of feces. The averages of the purity ratios were for 260/280, 1.93 ± 0.1, and for 260/230, 1.33 ± 0.36, which indicated that there was very little protein contamination and some chemical contamination remaining in some of the samples.

All qPCR data were manually curated, and the data that did not compile with standard requirements suggested by MIQE guidelines^15^ were excluded.

Primers targeting 10 of the 22 miRNAs (miR15a, miR16, miR21, miR26a, miR26b, mir192, miR200a‐3p, miR200c‐3p, let7a, and let7b) resulted in mono‐peaked melting curves and a PCR efficiency between 80% and 110%, and were, therefore, included for further analyses. The remaining 12 miRNAs did not show mono‐peaked melting curves and were not analyzed further.

### 3.3 Storage conditions at RT vs 4°C vs −20°C

No significant differences in the expression profiles of any of the miRNAs were seen when the samples were stored for 1 day at RT, 4°C, or *–*20°C (please see supporting information for graphical depiction; Figure S1). One cat had no data points for any of the three cDNA replicates for six of the miRNAs (miR‐15a, miR‐16, miR‐26a, miR‐26b, miR‐200a, and let‐7a), and for miR‐192 and let‐7b, the Cq was above 34 cycles and hence below the lower limit of quantitation in one or more of the triplicates from the feces stored at RT from day 1. The RT data from this cat was excluded from further analyses.

### 3.4 miRNA expression over time

**Figure 2:**
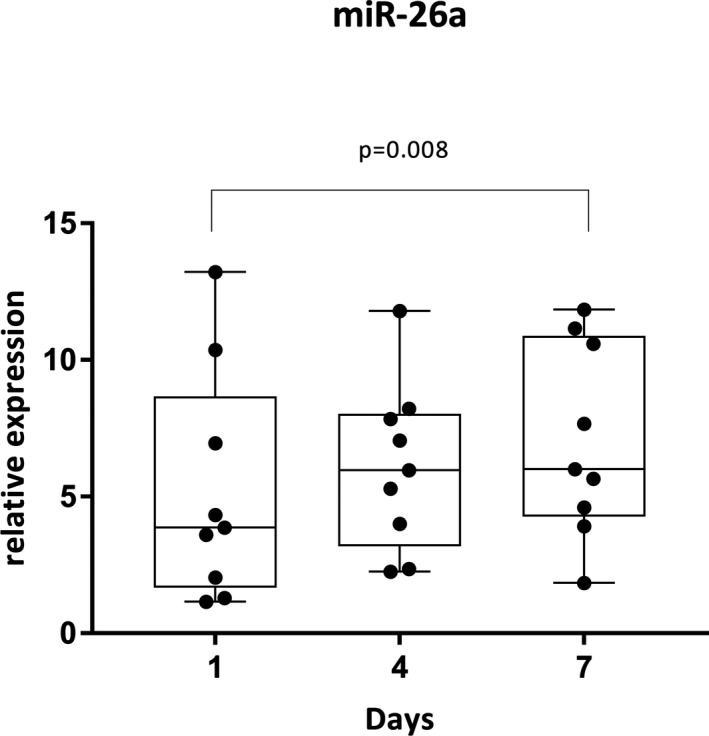
A box and whisker plot depicting the relative expression of fecal miR‐26a at days 1, 4, and 7 of collection. There is a significant difference in relative expression between days 1 and 7 of fecal collection. miR, microRNA

Only one miRNA out of nine analyzed, namely miR26a, showed a significant slight increase in expression (60% increase) between the frozen samples from the collection at days 1 and 7 (*P* = 0.0081; the fold change = 1.59 with a 95% confidence interval of 1.13, 2.26) as illustrated in Figure 2. There was no significant difference in the expression profiles of any of the other miRNAs when the samples from days 1, 4, and 7 and stored at –20°C were compared (Please see supporting information for graphical depiction, Figure S2).

## 4 DISCUSSION

This study is the first to identify fecal miRNAs in cats. Some of these miRNAs have been previously isolated in blood or tissue from cats or dogs,^8^, ^16^, ^17^ while others have been implicated in tumorigenesis in humans and mice,^18^, ^19^, ^20^ which makes them interesting targets for future analyses in cats with GI cancer.

Naturally evacuated feces are easily obtained by cat owners with indoor cats, but fecal collection still poses some practical challenges. Fecal consistency varies between bowel movements in cats, and the presence and amount of fur could influence the assay results. Even though we did not use it in the present study, the ratios from the spectrophotometric measurements could be used to correlate RNA purity with fecal consistency.

Three cDNA replicates from each RNA sample were analyzed, which improved the robustness of the results. Currently, there is a lack of standardization in the normalization of miRNA qPCR data, which could affect final data analyses. In this study, we normalized to the let‐7b miRNA, which was found to be the most stable of the analyzed miRNAs using two gold‐standard programs of normalization.^13^, ^14^ According to our experience, it is strongly recommended that each time a new tissue or treatment/condition is investigated, a new panel of reference genes are assessed.

No statistically significant difference between miRNA expression in samples stored for 24 hours at RT, 24 hours at 4°C, or directly at −20°C were identified. These results are in contrast to prior findings in feces from healthy dogs, where a significant difference was found in cfa‐miR‐16 and cfa‐miR‐21 stored at RT and −20°C, respectively.^8^ A sample (RT) from one cat was excluded because 8/10 data points were missing or Cq values were below the lower limit of quantitation. This sample had the lowest concentration of total RNA and lowest 260/230 ratio of all samples indicating problems in the RNA extraction procedure, which probably explains the poor qPCR results.

To the authors' knowledge, a systematic evaluation of stability from human feces has not been performed. However, it has been suggested that miRNAs are “relatively well‐preserved” in feces from healthy human controls and patients with CRC, who performed in‐home fecal collections and transported the sample at RT to the laboratory, although the timeframe was not further specified in this study.^21^ In this feline data, miRNA expression levels of nine miRNAs showed no significant differences between the three tested storage conditions indicating that miRNAs were clearly more stable compared with those of mRNAs.^22^

Nevertheless, for long‐term storage or analyses of other miRNAs not investigated in this study, we recommend freezing the samples as soon as possible at −20 or −80°C to ensure stable preservation. In general, storing fecal samples for miRNA expression studies identically to ensure minimal preanalytical variation would be beneficial.

Fecal miRNA expression was stable in the healthy cats for eight out of the nine miRNAs analyzed on the 3 days tested (days 1, 4, and 7). MiRNA‐26a showed significantly increased levels (60%, fold change = 1.59) between days 1 and 7, which could be explained by different fecal content compositions on day 7 compared with the rest of the samples for this cat, or it could indicate a real biologic increase in miR‐26a expression due to unknown physiologic reasons. Differences in dietary content would not explain these changes as each cat was fed the same diet during the study period. This miRNA was not different in any of the other comparisons between samples stored at different temperatures, frozen samples stored at days 1 and 4, or days 4 and 7. The miR‐26 family including miR‐26a and miR‐26b have been shown to be both tumor suppressors and to have oncogenic properties in different cancer types. Interestingly, healthy canine plasma and serum miR‐26a expression were significantly decreased after an increase in storage temperatures,^17^ which illustrates that the carrier matrix plays a vital role in miRNA preservation. In light of these results, we speculate that miR‐26a levels are more variable than other miRNAs. And, regarding the other miRNAs, it appears that a single fecal sample is sufficient to assess miRNA expression patterns in healthy cats. Since no studies have been performed in sick cats, it is unknown if miRNA stability will change or vary over time in these patients.

## DISCLOSURE

This project was funded by the Independent Research Fund Denmark and is part of a larger PhD project assessing fecal miRNA as biomarkers of GI disease in dogs and cats.
