# Supplementary figures and images for: Evaluation of fecal microRNA stability in healthy cats
Source: Vet Clin Pathol. 2019 Jun 26;48(3):455–60. doi: 10.1111/vcp.12757 (PMC6852515; doi:10.1111/vcp.12757)

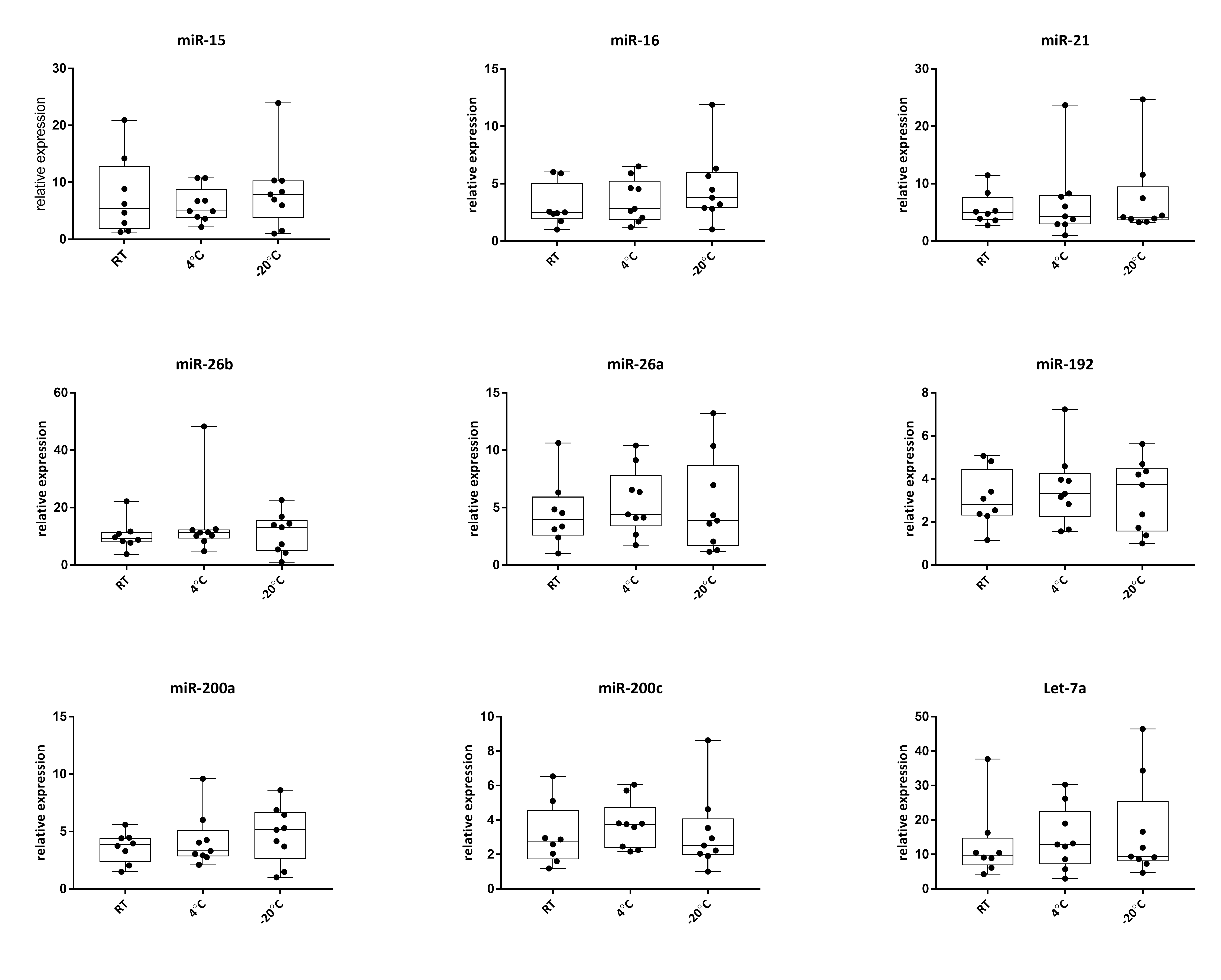

Supplement: Supplementary file 1 [file VCP-48-455-s001.tif]

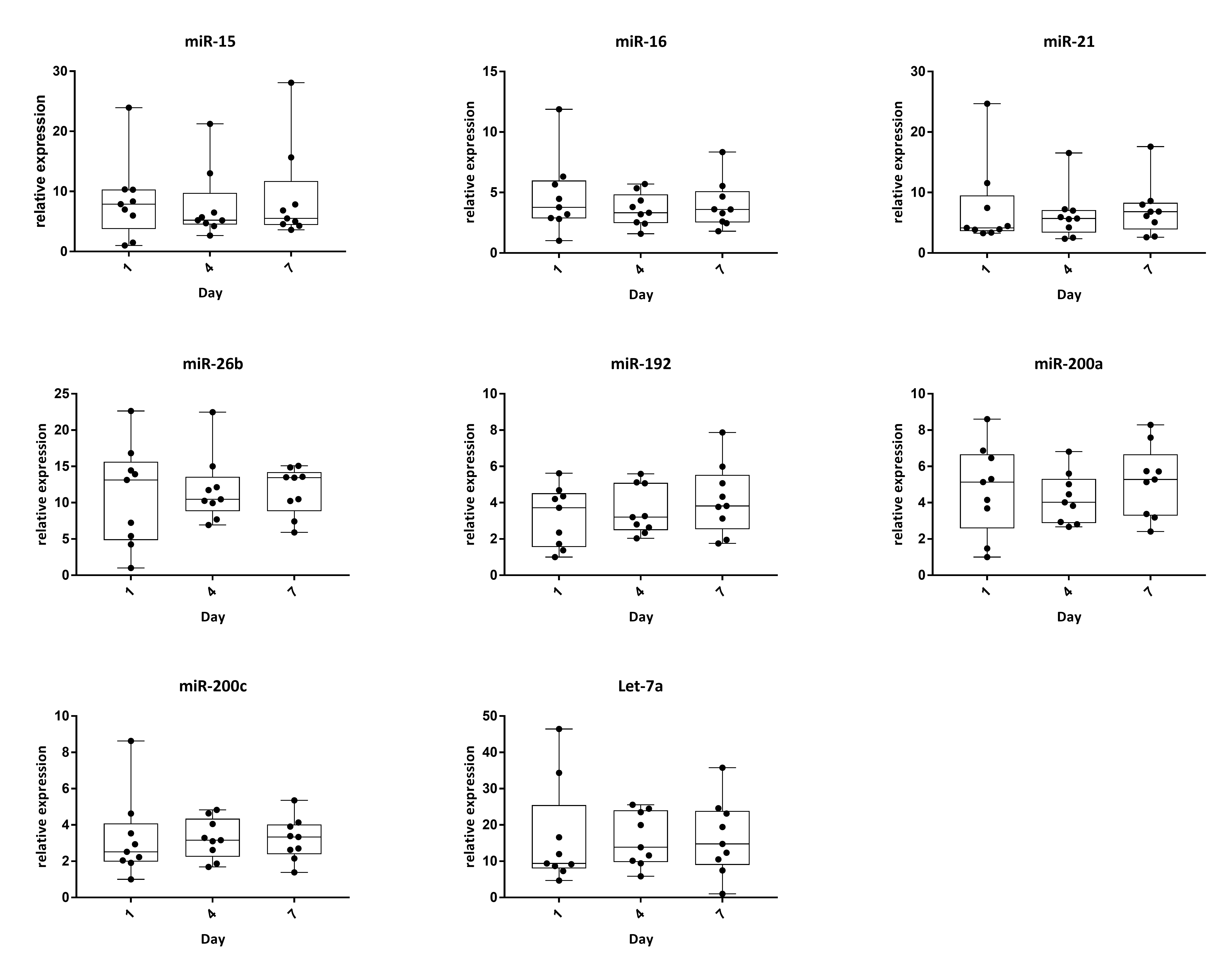

Supplement: Supplementary file 2 [file VCP-48-455-s002.tif]
