# Supplementary material for: Evaluation of fecal microRNA stability in healthy cats
Source: Vet Clin Pathol. 2019 Jun 26;48(3):455–60. doi: 10.1111/vcp.12757 (PMC6852515; doi:10.1111/vcp.12757)
Supplement: Supplementary file 3 [file VCP-48-455-s003.docx]

**Figure S1**. Relative expression of the nine analyzed miRs from healthy cats at different storage temperatures. No statistically significant differences in expression between temperatures were seen. RT, room temperature; miR, microRNA

**Figure S2**. Relative expression of the eight analyzed miRs from healthy cats. No statistically significant differences in expression between days 1, 4, and 7 were seen. miR, microRNA
